# The genome sequence of the Black-tipped Ermine, *Yponomeuta plumbella* (Denis & Schiffermüller, 1775)

**DOI:** 10.12688/wellcomeopenres.19368.1

**Published:** 2023-06-14

**Authors:** Douglas Boyes, Ian Sims, David Lees, Peter W.H. Holland, University of Oxford and Wytham Woods Genome Acquisition Lab, Natural History Museum Genome Acquisition Lab, Wellcome Sanger Institute Tree of Life programme, Wellcome Sanger Institute Scientific Operations: DNA Pipelines collective, Tree of Life Core Informatics collective, Darwin Tree of Life Consortium

**Affiliations:** 1 UK Centre for Ecology & Hydrology, Wallingford, England, UK; 2 The British Entomological and Natural History Society (BENHS), Reading, England, UK; 3 Natural History Museum, London, England, UK; 4 University of Oxford, Oxford, England, UK

**Keywords:** Yponomeuta plumbella, Black-tipped Ermine, genome sequence, chromosomal, Lepidoptera

## Abstract

We present a genome assembly from an individual male *Yponomeuta plumbella* (the Black-tipped Ermine; Arthropoda; Insecta; Lepidoptera; Yponomeutidae). The genome sequence is 636.6 megabases in span. Most of the assembly is scaffolded into 31 chromosomal pseudomolecules, including the Z sex chromosome. The mitochondrial genome has also been assembled and is 16.5 kilobases in length.

## Species taxonomy

Eukaryota; Metazoa; Ecdysozoa; Arthropoda; Hexapoda; Insecta; Pterygota; Neoptera; Endopterygota; Lepidoptera; Glossata; Ditrysia; Yponomeutoidea; Yponomeutidae; Yponomeutinae; *Yponomeuta; Yponomeuta plumbellus* (Denis & Schiffermüller, 1775) (NCBI:txid1594356).

## Background

The genus *Yponomeuta* contains several small moths with white forewings patterned with black speckles, collectively known as ‘small ermine’ moths. *Y. plumbella* (synonym *Y. plumbellus*), the Black-tipped Ermine or Large-spot Ermine, can be distinguished from similar species by a large black smudge halfway along the speckled forewing plus a dark mark at the wing apex ( Asher *et al.*, 2013). The ground colour of the wings is slightly off-white which may be the origin of the specific name *plumbella* meaning lead-like ( Emmet, 1991). *Y. plumbella* is widespread across northern, central and eastern Europe, southern parts of Scandinavia, southwest regions of Ireland, and central and southern counties of England and Wales ( GBIF Secretariat, 2023; Sterling & Parsons, 2018). In Britain, the moth is commonest on chalk-rich areas of southern England ( Sterling & Parsons, 2018). The moth has also been introduced to the United States with the first record being a 1949 specimen from Martha’s Vineyard, Massachusetts ( Hoebeke, 1987). It does not seem to be spreading from probable sites of introduction and has only been recorded from Massachusetts and Rhode Island ( MassMoths, 2023).

There has been confusion over whether *Y. plumbella* has one or two generations per year in Britain ( Agassiz, 1996; Kimber, 2023; Sterling & Parsons, 2018); records that report the life cycle stage are clearly consistent with a univoltine life cycle, with larvae found predominantly from April to June and adults in July and August ( Perry, 2023). The adults lay eggs on twigs of spindle *Euonymus europaeus* and the hatched larvae overwinter just below the empty eggshell. Development continues in spring with the larvae burrowing into fresh shoots, before spinning a silken web on the food plant where they live eating spindle leaves ( Agassiz, 1996). The webs spun by *Y. plumbella* are smaller and less conspicuous than those made by the spindle ermine *Y. cagnagella* on the same plant and contain just a few larvae ( Agassiz, 1996; Sterling & Parsons, 2018).

A genome sequence for *Y. plumbella* will facilitate research into the evolution of host plant specificity in herbivorous insects and contribute to the growing set of resources for studying the evolution of Lepidoptera.

### Genome sequence report

**Figure 1.**
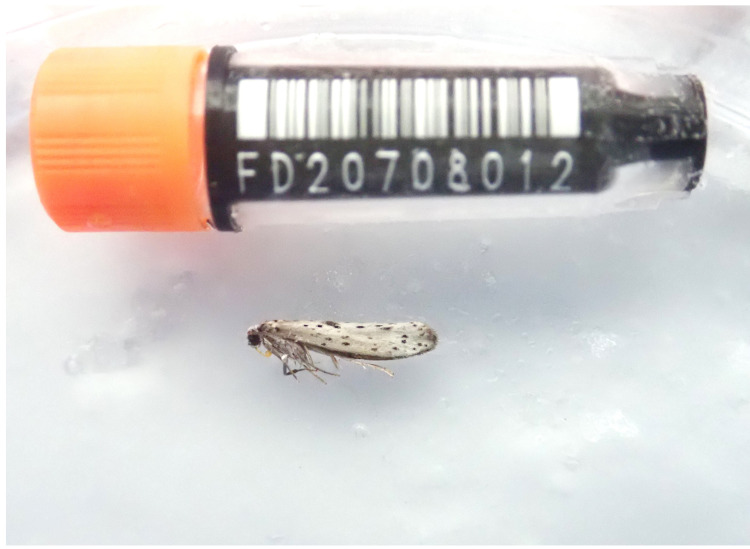
Photograph of the *Yponomeuta plumbella* (ilYpoPlub1) specimen used for genome sequencing.

The genome was sequenced from one male *Yponomeuta plumbella* ( Figure 1) collected from Wytham Woods, Oxfordshire (biological vice-county: Berkshire), UK (latitude 51.77, longitude –1.34). A total of 30-fold coverage in Pacific Biosciences single-molecule HiFi long reads was generated. Primary assembly contigs were scaffolded with chromosome conformation Hi-C data. Manual assembly curation corrected 10 missing joins or mis-joins, reducing the scaffold count by one. 

**Figure 2.**
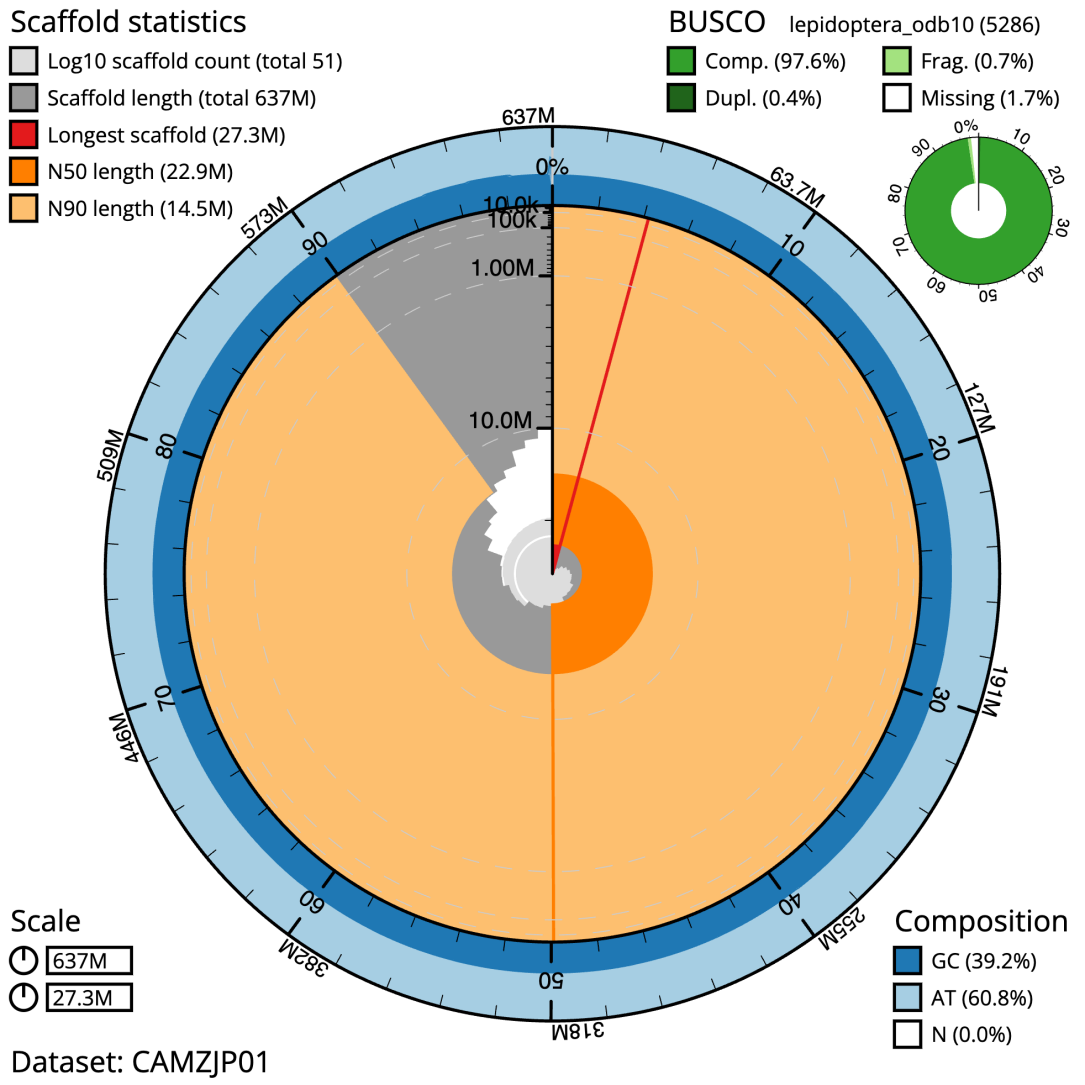
Genome assembly of *Yponomeuta plumbella*, ilYpoPlub1.1: metrics. The BlobToolKit Snailplot shows N50 metrics and BUSCO gene completeness. The main plot is divided into 1,000 size-ordered bins around the circumference with each bin representing 0.1% of the 636,643,948 bp assembly. The distribution of scaffold lengths is shown in dark grey with the plot radius scaled to the longest scaffold present in the assembly (27,348,352 bp, shown in red). Orange and pale-orange arcs show the N50 and N90 scaffold lengths (22,918,849 and 14,468,000 bp), respectively. The pale grey spiral shows the cumulative scaffold count on a log scale with white scale lines showing successive orders of magnitude. The blue and pale-blue area around the outside of the plot shows the distribution of GC, AT and N percentages in the same bins as the inner plot. A summary of complete, fragmented, duplicated and missing BUSCO genes in the lepidoptera_odb10 set is shown in the top right. An interactive version of this figure is available at https://blobtoolkit.genomehubs.org/view/ilYpoPlub1.1/dataset/CAMZJP01/snail.

The final assembly has a total length of 636.6 Mb in 51 sequence scaffolds with a scaffold N50 of 22.9 Mb ( Table 1). Most (99.81%) of the assembly sequence was assigned to 30 chromosomal-level scaffolds, representing 29 autosomes and the Z sex chromosome. Chromosome-scale scaffolds confirmed by the Hi-C data are named in order of size ( Figure 2– Figure 5; Table 2). ilYpoPlub1 is a male specimen, exhibiting the typical ZZ chromosome system. There is a possibility that chromosome 27 in this assembly may be an additional sex chromosome based on its alignment to 27Z in the assemblies of the female specimens of *Yponomeuta cagnagella* (GCA_947310995.1) and *Y. rorrellus* (GCA_947308005.1), both of which have been found to exhibit the sex chromosome trivalent system of *n* = 29A + A A ^W^ Z as described in ( Nilsson *et al.,* 1988). In the two female genome assemblies, the A component of the trivalent was assigned as chromosome 27Z based on its alignment to chromosome 27 in the closely related species *Y. sedellus* (ilYpoSedl1 GCA_934045075.1), a ZZ male specimen ( Boyes & Langdon, 2023).

**Table 1.** Genome data for *Yponomeuta plumbella*, ilYpoPlub1.1.

Project accession data		
Assembly identifier	ilYpoPlub1.1	
Species	*Yponomeuta plumbella*	
Specimen	ilYpoPlub1	
NCBI taxonomy ID	1594356	
BioProject	PRJEB55978	
BioSample ID	SAMEA7746626	
Isolate information	ilYpoPlub1, male (genome sequencing), ilYpoPlub2 (Hi-C scaffolding and RNA sequencing)	
Assembly metrics *		*Benchmark*
Consensus quality (QV)	63	*≥ 50*
*k*-mer completeness	100%	*≥ 95%*
BUSCO **	C:97.6%[S:97.2%,D:0.4%], F:0.7%,M:1.7%,n:5,286	*C ≥ 95%*
Percentage of assembly mapped to chromosomes	99.81%	*≥ 95%*
Sex chromosomes	Z chromosome	*localised homologous pairs*
Organelles	Mitochondrial genome assembled	*complete single alleles*
Raw data accessions		
PacificBiosciences SEQUEL II	ERR10224915, ERR10224916	
Hi-C Illumina	ERR10297811	
PolyA RNA-Seq Illumina	ERR10378033	
Genome assembly		
Assembly accession	GCA_947310845.1	
*Accession of alternate haplotype*	GCA_947310575.1	
Span (Mb)	636.6	
Number of contigs	66	
Contig N50 length (Mb)	21.0	
Number of scaffolds	51	
Scaffold N50 length (Mb)	22.9	
Longest scaffold (Mb)	27.4	

* Assembly metric benchmarks are adapted from column VGP-2020 of “Table 1: Proposed standards and metrics for defining genome assembly quality” from ( Rhie *et al.*, 2021). ** BUSCO scores based on the lepidoptera_odb10 BUSCO set using v5.3.2. C = complete [S = single copy, D = duplicated], F = fragmented, M = missing, n = number of orthologues in comparison. A full set of BUSCO scores is available at https://blobtoolkit.genomehubs.org/view/ilYpoPlub1.1/dataset/CAMZJP01/busco.

**Figure 3.**
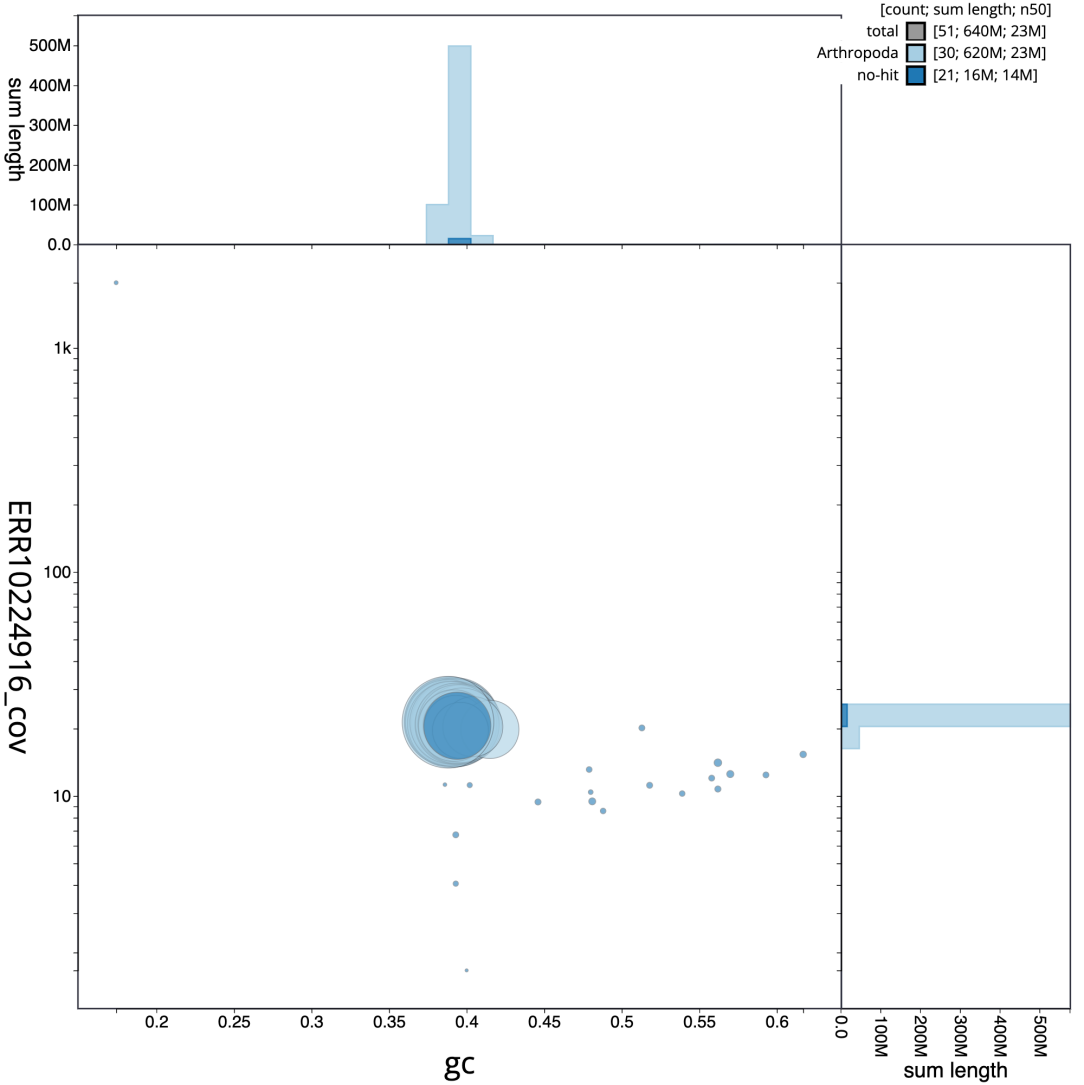
Genome assembly of *Yponomeuta plumbella*, ilYpoPlub1.1: BlobToolKit GC-coverage plot. Scaffolds are coloured by phylum. Circles are sized in proportion to scaffold length. Histograms show the distribution of scaffold length sum along each axis. An interactive version of this figure is available at https://blobtoolkit.genomehubs.org/view/ilYpoPlub1.1/dataset/CAMZJP01/blob.

**Figure 4.**
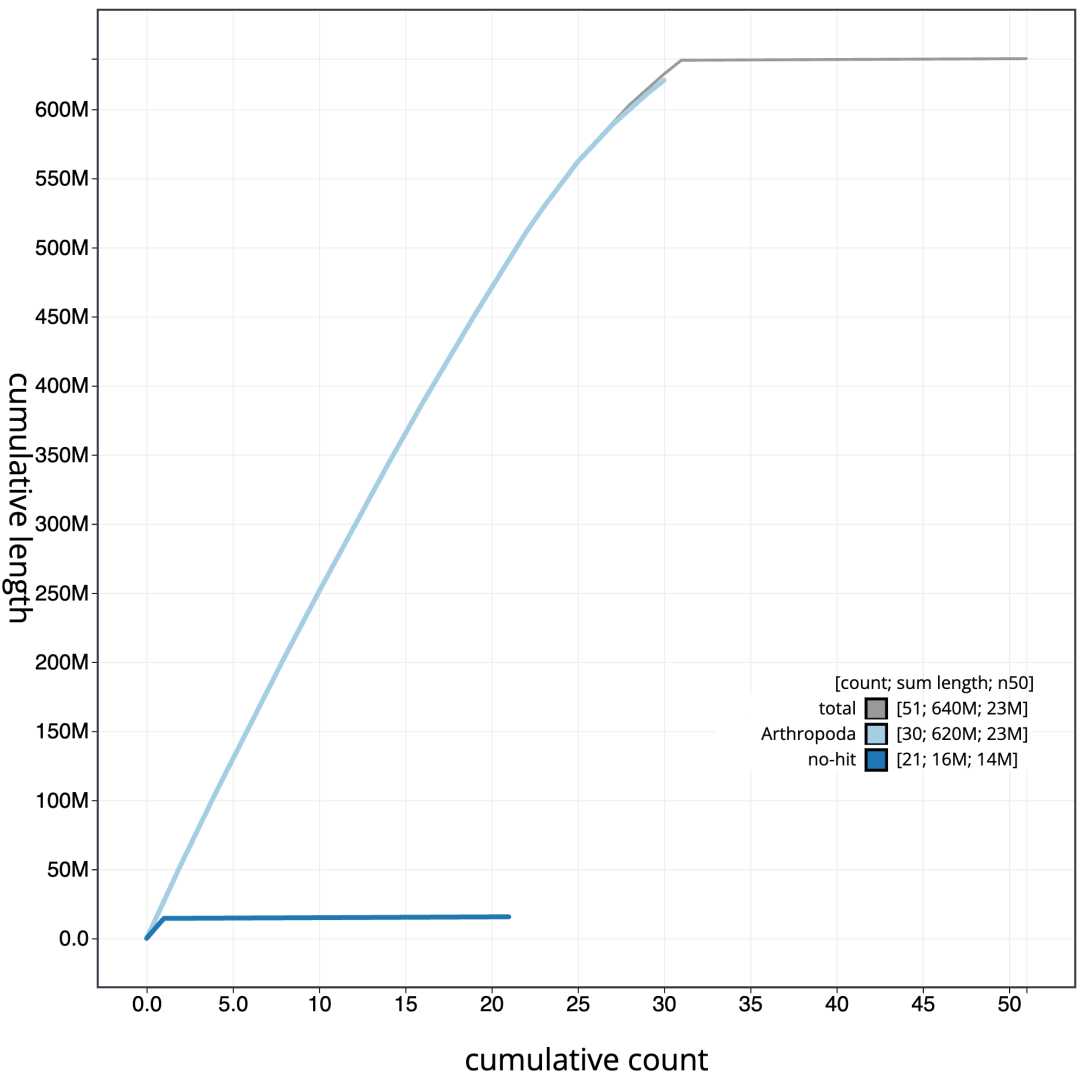
Genome assembly of *Yponomeuta plumbella*, ilYpoPlub1.1: BlobToolKit cumulative sequence plot. The grey line shows cumulative length for all scaffolds. Coloured lines show cumulative lengths of scaffolds assigned to each phylum using the buscogenes taxrule. An interactive version of this figure is available at https://blobtoolkit.genomehubs.org/view/ilYpoPlub1.1/dataset/CAMZJP01/cumulative.

**Figure 5.**
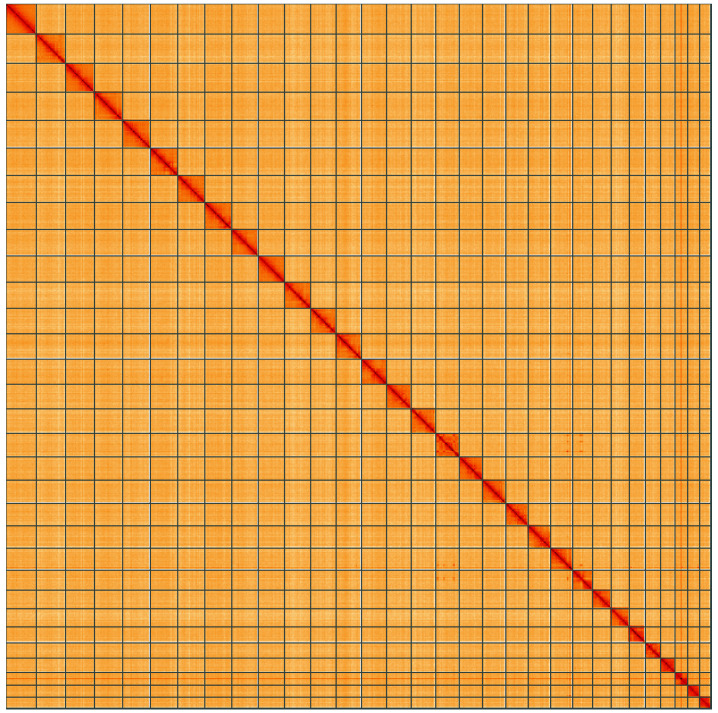
Genome assembly of *Yponomeuta plumbella*, ilYpoPlub1.1: Hi-C contact map of the ilYpoPlub1.1 assembly, visualised using HiGlass. Chromosomes are shown in order of size from left to right and top to bottom. An interactive version of this figure may be viewed at https://genome-note-higlass.tol.sanger.ac.uk/l/?d=YSKqph9NR0CkSw-LVD24Vg.

**Table 2.** Chromosomal pseudomolecules in the genome assembly of *Yponomeuta plumbella*, ilYpoPlub1.

INSDC accession	Chromosome	Size (Mb)	GC%
OX371192.1	1	26.37	39
OX371193.1	2	26.08	38.9
OX371194.1	3	23.82	39
OX371195.1	4	25.43	39.2
OX371196.1	5	24.4	38.8
OX371197.1	6	24.98	38.8
OX371198.1	7	24.65	39.3
OX371199.1	8	24.37	39.3
OX371200.1	9	23.58	38.8
OX371201.1	10	22.92	39.1
OX371202.1	11	23.73	39.3
OX371203.1	12	22.62	39.2
OX371204.1	13	22.17	39.1
OX371205.1	14	22.97	38.9
OX371206.1	15	21.22	39.3
OX371207.1	16	22.12	39
OX371208.1	17	21.02	39.4
OX371209.1	18	19.92	39.2
OX371210.1	19	20.95	39.3
OX371211.1	20	20.22	39.7
OX371212.1	21	20.16	39.4
OX371213.1	22	16.8	39.2
OX371214.1	23	17.95	39.7
OX371215.1	24	16.35	39.2
OX371216.1	25	14.47	39.4
OX371217.1	26	13.71	39.3
OX371218.1	27	12.97	39.3
OX371219.1	28	10.78	41.5
OX371220.1	29	11.48	40.4
OX371221.1	30	9.97	39.6
OX371191.1	Z	27.35	38.8
OX371222.1	MT	0.02	17.5

While not fully phased, the assembly deposited is of one haplotype. Contigs corresponding to the second haplotype have also been deposited. The mitochondrial genome was also assembled and can be found as a contig within the multifasta file of the genome submission.

The estimated Quality Value (QV) of the final assembly is 63 with *k*-mer completeness of 100%, and the assembly has a BUSCO v5.3.2 completeness of 97.6% (single = 97.2%, duplicated = 0.4%), using the lepidoptera_odb10 reference set ( *n* = 5,286).

Metadata for specimens, spectral estimates, sequencing runs, contaminants and pre-curation assembly statistics can be found at https://links.tol.sanger.ac.uk/species/1594356.

## Methods

### Sample acquisition and nucleic acid extraction

A male *Yponomeuta plumbella* specimen (ilYpoPlub1) was collected from Wytham Woods, Oxfordshire (biological vice-county: Berkshire), UK (latitude 51.77, longitude –1.34) on 1 August 2020. The specimen was taken from woodland habitat by Douglas Boyes (University of Oxford) using a light trap. The specimen was identified by the collector and snap-frozen on dry ice. This specimen was used for genome sequencing.

A second male *Yponomeuta plumbella* specimen (ilYpoPlub2) was collected in Hartslock Nature Reserve, UK (latitude 51.51, longitude –1.11) on 29 July 2021 by Ian Sims (British Entomological and Natural History Society) using a light trap. This specimen was identified by David Lees (Natural History Museum) and dry frozen at –80°C. The specimen ilYpoPlub2 was used for Hi-C scaffolding.

DNA was extracted at the Tree of Life laboratory, Wellcome Sanger Institute (WSI). The ilYpoPlub1 sample was weighed and dissected on dry ice with tissue set aside for Hi-C sequencing. Whole organism tissue was using a Nippi Powermasher fitted with a BioMasher pestle. High molecular weight (HMW) DNA was extracted using the Qiagen MagAttract HMW DNA extraction kit. HMW DNA was sheared into an average fragment size of 12–20 kb in a Megaruptor 3 system with speed setting 30. Sheared DNA was purified by solid-phase reversible immobilisation using AMPure PB beads with a 1.8X ratio of beads to sample to remove the shorter fragments and concentrate the DNA sample. The concentration of the sheared and purified DNA was assessed using a Nanodrop spectrophotometer and Qubit Fluorometer and Qubit dsDNA High Sensitivity Assay kit. Fragment size distribution was evaluated by running the sample on the FemtoPulse system.

RNA was extracted from abdomen tissue of ilYpoPlub2 in the Tree of Life Laboratory at the WSI using TRIzol, according to the manufacturer’s instructions. RNA was then eluted in 50 μl RNAse-free water and its concentration assessed using a Nanodrop spectrophotometer and Qubit Fluorometer using the Qubit RNA Broad-Range (BR) Assay kit. Analysis of the integrity of the RNA was done using Agilent RNA 6000 Pico Kit and Eukaryotic Total RNA assay.

### Sequencing

Pacific Biosciences HiFi circular consensus DNA sequencing libraries were constructed according to the manufacturers’ instructions. Poly(A) RNA-Seq libraries were constructed using the NEB Ultra II RNA Library Prep kit. DNA and RNA sequencing were performed by the Scientific Operations core at the WSI on Pacific Biosciences SEQUEL II (HiFi) and Illumina NovaSeq 6000 (RNA-Seq) instruments. Hi-C data were also generated from thorax tissue of ilYpoPlub2 using the Arima v2 kit and sequenced on the Illumina NovaSeq 6000 instrument.

### Genome assembly, curation and evaluation

Assembly was carried out with Hifiasm ( Cheng *et al.*, 2021) and haplotypic duplication was identified and removed with purge_dups ( Guan *et al.*, 2020). The assembly was then scaffolded with Hi-C data ( Rao *et al.*, 2014) using YaHS ( Zhou *et al.,* 2023). The assembly was checked for contamination as described previously ( Howe *et al.*, 2021). Manual curation was performed using HiGlass ( Kerpedjiev *et al.*, 2018) and Pretext ( Harry, 2022). The mitochondrial genome was assembled using MitoHiFi ( Uliano-Silva *et al.*, 2022), which runs MitoFinder ( Allio *et al.*, 2020) or MITOS ( Bernt *et al.*, 2013) and uses these annotations to select the final mitochondrial contig and to ensure the general quality of the sequence. To evaluate the assembly, MerquryFK was used to estimate consensus quality (QV) scores and *k*-mer completeness ( Rhie *et al.*, 2020). The genome was analysed within the BlobToolKit environment ( Challis *et al.*, 2020) and BUSCO scores ( Manni *et al.*, 2021; Simão *et al.*, 2015) were calculated. Table 3 contains a list of software tool versions and sources.

**Table 3.** Software tools: versions and sources.

Software tool	Version	Source
BlobToolKit	4.0.7	https://github.com/blobtoolkit/blobtoolkit
BUSCO	5.3.2	https://gitlab.com/ezlab/busco
Hifiasm	0.16.1-r375	https://github.com/chhylp123/hifiasm
HiGlass	1.11.6	https://github.com/higlass/higlass
Merqury	MerquryFK	https://github.com/thegenemyers/MERQURY.FK
MitoHiFi	2	https://github.com/marcelauliano/MitoHiFi
PretextView	0.2	https://github.com/wtsi-hpag/PretextView
purge_dups	1.2.3	https://github.com/dfguan/purge_dups
YaHS	yahs-1.1.91eebc2	https://github.com/c-zhou/yahs

### Ethics and compliance issues

The materials that have contributed to this genome note have been supplied by a Darwin Tree of Life Partner. The submission of materials by a Darwin Tree of Life Partner is subject to the Darwin Tree of Life Project Sampling Code of Practice. By agreeing with and signing up to the Sampling Code of Practice, the Darwin Tree of Life Partner agrees they will meet the legal and ethical requirements and standards set out within this document in respect of all samples acquired for, and supplied to, the Darwin Tree of Life Project. All efforts are undertaken to minimise the suffering of animals used for sequencing. Each transfer of samples is further undertaken according to a Research Collaboration Agreement or Material Transfer Agreement entered into by the Darwin Tree of Life Partner, Genome Research Limited (operating as the Wellcome Sanger Institute), and in some circumstances other Darwin Tree of Life collaborators.

## Data Availability

European Nucleotide Archive: *Yponomeuta plumbellus* (black-tipped ermine). Accession number PRJEB55978; https://identifiers.org/ena.embl/PRJEB55978. ( Wellcome Sanger Institute, 2022) The genome sequence is released openly for reuse. The *Yponomeuta plumbella* genome sequencing initiative is part of the Darwin Tree of Life (DToL) project. All raw sequence data and the assembly have been deposited in INSDC databases. The genome will be annotated using available RNA-Seq data and presented through the Ensembl pipeline at the European Bioinformatics Institute. Raw data and assembly accession identifiers are reported in Table 1.

## Author information

Members of the University of Oxford and Wytham Woods Genome Acquisition Lab are listed here: https://doi.org/10.5281/zenodo.4789928.

Members of the Natural History Museum Genome Acquisition Lab are listed here: https://doi.org/10.5281/zenodo.4790043.

Members of the Darwin Tree of Life Barcoding collective are listed here: https://doi.org/10.5281/zenodo.4893703.

Members of the Wellcome Sanger Institute Tree of Life programme are listed here: https://doi.org/10.5281/zenodo.4783585.

Members of Wellcome Sanger Institute Scientific Operations: DNA Pipelines collective are listed here: https://doi.org/10.5281/zenodo.4790455.

Members of the Tree of Life Core Informatics collective are listed here: https://doi.org/10.5281/zenodo.5013541.

Members of the Darwin Tree of Life Consortium are listed here: https://doi.org/10.5281/zenodo.4783558.
