# Supplementary material for: The genome sequence of the Black-tipped Ermine, Yponomeuta plumbella (Denis & Schiffermüller, 1775)
Source: Wellcome Open Res. 2023 Jun 14;8:246. [Version 1] doi: 10.12688/wellcomeopenres.19368.1 (PMC10690038; doi:10.12688/wellcomeopenres.19368.1)
Supplement: Peer review history

# Reviewer report: Reviewer response for version 1

**DOI:** 10.21956/wellcomeopenres.21455.r70064

**Published:** 2023-11-30

**Authors:** Ilik J Saccheri

**Affiliations:** 1 University of Liverpool, Liverpool, England, UK

Please clarify why the abstract refers to 31 chromosomal pseudomolecules (incl. Z), but the second paragraph of the ‘Genome sequence report’ section, specifies ‘30 chromosomal-level scaffolds, representing 29 autosomes and the Z’? Figure 5 has 31 scaffolds. Is it that one of them is not ‘chromosome-level’ but somehow is considered a ‘chromosomal pseudomolecule’?

Are sufficient details of methods and materials provided to allow replication by others?

Yes

Is the rationale for creating the dataset(s) clearly described?

Yes

Are the datasets clearly presented in a useable and accessible format?

Yes

Are the protocols appropriate and is the work technically sound?

Yes

Reviewer Expertise:

evolutionary ecology and lepidopteran genetics/genomics

I confirm that I have read this submission and believe that I have an appropriate level of expertise to confirm that it is of an acceptable scientific standard.

# Reviewer report: Reviewer response for version 1

**DOI:** 10.21956/wellcomeopenres.21455.r70060

**Published:** 2023-11-30

**Authors:** Xiangyu Hao

**Affiliations:** 1 Northwest A&F University, Yangling, Shaanxi, China

The manuscript detailing the genome assembly of *Yponomeuta plumbella* presents a comprehensive and meticulously curated assembly process, offering valuable insights into Lepidoptera genetics. The 636.6 Mb assembly, scaffolded into 31 chromosomal pseudomolecules, including the Z sex chromosome,  follows the standards established by the Darwin Tree of Life.

 The study's methodology, encompassing diverse sequencing techniques and detailed assembly curation, ensures the robustness and credibility of the genomic resource. The provided metadata and transparency in methodology enhance the study's reproducibility.

 The manuscript's contextualization of the genome sequence's significance in understanding the evolution of host plant specificity in herbivorous insects within Lepidoptera, along with insights into *Y. plumbella*'s life cycle in Britain, highlights its ecological importance.

Are sufficient details of methods and materials provided to allow replication by others?

Yes

Is the rationale for creating the dataset(s) clearly described?

Yes

Are the datasets clearly presented in a useable and accessible format?

Yes

Are the protocols appropriate and is the work technically sound?

Yes

Reviewer Expertise:

Zoology, Entomology, Evolutionary Biology

I confirm that I have read this submission and believe that I have an appropriate level of expertise to confirm that it is of an acceptable scientific standard.
